# Body Asymmetry and Sports Specialization: An Exploratory Anthropometric Comparison of Adolescent Canoeists and Kayakers

**DOI:** 10.3390/jfmk10010070

**Published:** 2025-02-20

**Authors:** Carlos Abraham Herrera-Amante, William Carvajal-Veitía, Rodrigo Yáñez-Sepúlveda, Fernando Alacid, Juan Gavala-González, José Francisco López-Gil, Jorge Olivares-Arancibia, César Octavio Ramos-García

**Affiliations:** 1 Nutritional Assessment and Nutritional Care Laboratory (LECEN), Division of Health Sciences, Tonalá University Center, University of Guadalajara (UdeG), Tonalá 45425, Mexico; carlos.amante@academicos.udg.mx; 2 Research Division, Ibero-American Institute of Sports Sciences and Human Movement (IICDEM), Guadalajara 44510, Mexico; 3 Ibero-American Network of Researchers in Applied Anthropometry (RIBA^2^), 04120 Almería, Spain; wiliamcarvajal790@gmail.com (W.C.-V.); falacid@ual.es (F.A.); jgavala@us.es (J.G.-G.); 4 Institute of Sports Medicine (IMD), Havana 10800, Cuba; 5 Faculty of Education and Social Sciences, Universidad Andres Bello, Viña del Mar 2520000, Chile; rodrigo.yanez.s@unab.cl; 6 Department of Education, Health Research Centre, University of Almería (UAL), 04120 Almeria, Spain; 7 Department of Physical Education and Sports, Universidad de Sevilla (US), 41013 Sevilla, Spain; 8 One Health Research Group, Universidad de Las Américas, Quito 170124, Ecuador; 9 Grupo AFySE, Investigación en Actividad Física y Salud Escolar, Escuela de Pedagogía en Educación Física, Facultad de Educación, Universidad de las Américas, Santiago 7500975, Chile; jolivares@udla.cl

**Keywords:** water sports, anthropometry, body composition, athletic injuries, muscle imbalances, bilateral deficits

## Abstract

**Background/Objectives:** The evaluation of body asymmetry between the right and left sides of the body is crucial in the context of sports because of its potential impact on performance and injury prevention. This study analyzed the anthropometric differences between the right and left sides of the body in adolescent (13.0 [2.0] years) male canoeists and kayakers from Spain. This study aimed to explore the asymmetries associated with these disciplines. **Methods**: Anthropometric assessments were carried out on 27 male adolescents (13 canoeists and 14 kayakers). A total of 58 anthropometric variables were assessed, including 23 pairs of bilateral variables and 12 unilateral measurements. The evaluations included basic measures, skinfolds, girths, lengths, and breadths. Relative differences between sides were calculated via the bilateral asymmetry index (%BAI). Nonparametric tests, such as the Wilcoxon signed-rank test for within-group comparisons and the Mann–Whitney U test for between-group comparisons, were applied. **Results**: Comparisons between body sides within the groups revealed significant asymmetries in the subscapular skinfold (*p* = 0.010) in canoeists and in the mid-thigh girth (*p* = 0.041) in kayakers. Among the groups, differences were found in the subscapular skinfold (*p* = 0.010) and the bicep skinfold (*p* = 0.038) on the right side. **Conclusions**: Although significant differences were found in some variables, no distinctive profile of the differences between canoeists and kayakers was established in the categories analyzed. These results suggest that, in general, body asymmetries do not significantly distinguish between these disciplines within the sample studied. Further research is needed to better understand the implications of these differences for performance and injury prevention in specific adolescent sports contexts.

## 1. Introduction

Symmetry is defined as the quality of an object that exhibits exact equality in size, shape, and structure across its two halves when divided along an axis [1]. In contrast, asymmetry refers to variations in characteristics between one side of the body and the other and can be influenced by genetic and environmental factors and the differential use of body segments [2,3]. In the sports domain, the study of body symmetry is highly relevant, as it provides valuable information for performance evaluations, development and maturation, injury prevention, training optimization, and equipment innovation [1,4,5,6]. Sports with unilateral gestures, such as tennis, rowing, and fencing, often induce notable asymmetries due to their repetitive motion patterns, which differ significantly from those in bilateral or symmetrical sports such as swimming or gymnastics [1,7]. These differences underscore the need to understand asymmetries across sports disciplines.

Canoeing and kayaking are two paddle sports that, despite their similarities, differ significantly in terms of their biomechanics and muscular demands. Canoeing (specifically Canadian canoeing) requires paddlers to kneel on one knee while executing unilateral strokes on one side of the body using a single-bladed paddle. This repetitive motion predominantly engages the upper body, particularly the deltoids, latissimus dorsi, and trapezius, while also placing asymmetric demands on the core and lower limbs due to the required kneeling position [8]. Recent research has demonstrated the importance of assessing asymmetries in athletes, particularly in sports like canoeing, where repetitive movements may lead to imbalances [9].

In contrast, kayaking is performed in a seated position with the legs extended forward and the use of a double-bladed paddle, which allows for alternating strokes on both sides of the body. This movement pattern generally distributes muscular effort more symmetrically across the upper body, engaging the pectorals, latissimus dorsi, and obliques, while still requiring strong lower-body stabilization [8]. However, despite its more balanced movement mechanics, kayaking can still lead to asymmetries, particularly in terms of muscle activation patterns and joint loading over time.

Among the various methodologies for evaluating body symmetry, anthropometry stands out as a valuable and highly reproducible technique. Its ability to enable bilateral and segmental analyses allows for meaningful insights into the impact of body asymmetries [10]. However, anthropometric assessments have traditionally focused on the right side of the body, as international standards, such as those established by the International Society for the Advancement of Kinanthropometry (ISAK), prioritize right-side measurements in their protocols [11]. This approach may overlook the importance of systematically monitoring asymmetries to better understand their practical implications during training and in competitive settings. Furthermore, recent advancements have highlighted the potential of three-dimensional scanning technologies and advanced imaging tools in capturing more detailed asymmetry metrics, which could complement traditional anthropometry [12]. While these tools offer precision, they remain underutilized in youth sports research.

Sports such as canoeing and kayaking are particularly intriguing in the context of asymmetry, as their movements impose specific biomechanical demands that influence muscular and skeletal development [8]. Studies have shown that paddlers often develop more significant asymmetries than other athletes do in bilateral sports, such as swimming, primarily because of the repetitive and unidirectional forces exerted during paddling [13,14]. These sport-specific asymmetries may affect performance and play an important role in injury susceptibility, especially during adolescent growth spurts, as athletes experience rapid physical changes [15].

Examining asymmetries could help elucidate their potential impact on the development of different sports modalities. Research has shown significant differences in anthropometric dimensions, such as body girths, breadths, and lengths, favoring the dominant side of the body [16,17]. Furthermore, the effects of differences in body composition symmetry on performance have been documented [18]. However, some studies have not found an association between training volume and the magnitude of asymmetry [7], while other studies identify training as having a strong influence [5,19,20]. The divergence in these findings suggests that the relationship between asymmetry and performance is complex and multifactorial and could be influenced by age, training intensity, and duration [18,21]. These varying results underscore the need for more research to understand how training impacts symmetry and how these effects may differ across sports modalities. Currently, there is limited information on what levels of asymmetry might be beneficial or detrimental to athletic performance [16]. A recent study on kettlebell athletes found notable symmetry in their body composition and strength, particularly in the upper body, suggesting that training characteristics may play a key role in mitigating asymmetries. These findings contribute to the ongoing debate on the relationship between asymmetry and performance, highlighting the need for further research that considers factors such as age, level of competition, and type and amount of training [3].

Beyond their implications for performance, addressing asymmetries in adolescent athletes may provide broader benefits regarding their long-term health and injury prevention. During adolescence, physical growth occurs at an accelerated pace, creating a critical window where muscle imbalances and joint stresses may lead to chronic issues if left unaddressed [22]. Early intervention programs incorporating an asymmetry assessment could guide coaches and healthcare professionals in designing tailored training regimens. By identifying and mitigating excessive asymmetries, such initiatives could enhance biomechanical efficiency while reducing the likelihood of the overuse injuries common in water sports [15,20].

One less explored aspect of applied anthropometry is the difference in the symmetry of anthropometric profiles across sports modalities. Studies in Sports Science have focused primarily on physical capacity and strength rather than body composition. A study by Stagi and associates [3] examined the relationship between symmetry, body composition, and physical performance, contributing to the broader discussion on how asymmetry may influence athletic outcomes. However, several questions remain unanswered: do differences in the symmetry of anthropometric profiles correspond to the motor performance demands of each sport modality? What trends are characteristic of each sport? In which body region is asymmetry more pronounced, the upper or lower body? Previous findings indicate that asymmetries may evolve over time due to sport-specific demands, with younger athletes often showing less pronounced differences than seasoned competitors do [23,24]. The dynamic nature of asymmetries underscores the importance of longitudinal studies within sports research.

The literature suggests that most sports exhibit pronounced functional asymmetries due to their specific demands [13,21]. However, the classification of sports as symmetrical or asymmetrical is based mainly on their motor gestures. In practice, all athletes exhibit asymmetries within a generally permissible range. For example, Canadian canoeing and kayaking have been classified as asymmetrical [13,23] and symmetrical [13], respectively. However, there is insufficient evidence to determine whether the asymmetrical or symmetric load in each sport is reflected in a specific anthropometric profile or if it varies by competitive age group [23]. Other studies have also suggested that certain degrees of asymmetry, particularly in strength and muscle mass, may confer competitive advantages in asymmetric sports, while excessive imbalances increase the risk of injury [25,26]. In this context, Krzykała and associates [13] noted that during the biological development of young canoe athletes, specialized training could cause asymmetries, leading to differences in muscle mass between sides. However, prolonged training appears to reduce lower-limb muscle mass asymmetry among older competitors [23].

Furthermore, a few studies have highlighted the differences between kayak paddlers and canoe paddlers, which have been attributed to the continuous physical development that kayakers require to stay competitive and the need for young canoeists to focus more intensively on refining their technical skills [8,15]. Given the limited literature on adolescent paddlers, this study fills a critical gap by exploring these dynamics at a developmental stage, where interventions may yield significant long-term benefits [20,27]. This research addresses these discrepancies by comparing the differences in the symmetry of the anthropometric profiles of Spanish adolescents practicing Canadian canoeing and those practicing kayaking. The findings of this study could enhance the application of anthropometry as, despite its high reproducibility, its use in profiling symmetry differences has been limited. This underutilization stems from the small number of anthropometric indicators typically employed [1,5,19,20,28] or the reliance on alternative methods such as dual-energy X-ray absorptiometry (DXA), bioelectrical impedance analysis (BIA), or other body composition studies [2,3,13,18,21]. Our hypothesis posits that asymmetries in young practitioners are more pronounced in sports disciplines such as Canadian canoeing compared to kayaking.. By addressing this hypothesis, this paper aims to inform coaches, sports scientists, and medical professionals about the complex relationship between training regimens and asymmetry, ultimately contributing to optimized performance and injury prevention strategies [29].

## 2. Materials and Methods

### 2.1. Study Design

A cross-sectional descriptive study was designed. The participants reported to the testing area where data collection was conducted only once. The Strengthening the Reporting of Observational Studies in Epidemiology (STROBE) criteria for cross-sectional research were followed in the design of this study [30,31].

### 2.2. Setting

This study was conducted in Seville, Spain. Individual information (demographics, descriptive data, sports discipline, and experience) was collected after a brief questionnaire was completed. Signed parental consent was obtained from the parents or legal guardians of all participants. Parents and study participants were fully and appropriately informed about the participation requirements and the purpose, risks, and benefits of the study. All measurements were taken in the presence of other athletes and their coaches to ensure a comfortable and familiar environment for the participants. This study was carried out in accordance with the ethical principles for medical research outlined in the international guidelines for good clinical practice and the Declaration of Helsinki [32]. The Institutional Ethical Committee of the University of Murcia approved this study (SKMBT-C25211110314021).

### 2.3. Participants

Anthropometric data from 27 adolescent Spanish males (13 canoeists and 14 kayakers), with a median age of 13.0 years (IQR: 2.0) for both groups, were analyzed. The anthropometric data were collected during the preseason period and include data from athletes from three Sevillian clubs who met the necessary standards to compete in their category in national championships. Athletes who met the following inclusion criteria were invited to participate in this study: (i) those who attended at least 90% of training sessions and (ii) had a performance level that allowed them to compete in national championships in their category. The exclusion criteria included (i) not providing written consent (parental consent) for the procedures to be conducted or data to be disclosed for research purposes at the time of the evaluations.

Arriving at the assessment area without appropriate clothing was considered a removal criterion.

### 2.4. Variables

A total of 58 anthropometric variables were assessed, including 23 pairs of bilateral variables and 12 unilateral measurements, following the guidelines established by the International Society for the Advancement of Kinanthropometry (ISAK) [11]. All anthropometric measurements were taken two or three times (with a third measurement taken if the difference between the first two measurements exceeded 5% for skinfolds and 1% in the remaining measures), and the mean or the median value was used for data analysis, respectively. The technical error of measurement (TEM) was calculated according to Pederson and Gore [33].

### 2.5. Measurements

Anthropometric measurements were performed based on the international standards established by the ISAK [11]. These protocols are specifically designed for the evaluation of the right side of the body; however, they were adapted in this study to assess both sides. Measurements were carried out by two certified anthropometrists: one level 3 (ISAK L3) and one level 2 (ISAK L2) anthropometrist. The level 3 anthropometrist, as they were the most experienced, conducted all the measurements, while the level 2 anthropometrist assisted and recorded the data.

Body mass (kg) was determined using a digital scale with a precision of 50 g (SECA^®^ 874, Hamburg, Germany). Stature (cm) and sitting height (cm) were assessed with a 1 mm precision stadiometer (SECA^®^ 217, Hamburg, Germany). Skinfold thickness (mm) was measured with a skinfold caliper with a precision of 0.2 mm (Harpenden, British Indicators, Crymych, UK). Girths (cm) were measured with a flexible, nonstretchable metal tape with a precision of 1 mm (SmartMet Kinanthropometric Assessment^®^, Jalisco, Mexico). Lengths (cm) and breadths (cm) were measured using a segmentometer and large bone caliper with a precision of 1 mm (SmartMet Kinanthropometric Assessment^®^, Jalisco, Mexico). All instruments were calibrated before the evaluations to minimize measurement errors.

### 2.6. Statistical Methods

Statistical analysis was performed in R Studio version 4.4.1, and we evaluated 46 anthropometric variables measured on both sides of the body (23 variables on the right side and 23 on the left side). Relative differences between measurements on the left and right sides of the body were calculated using the bilateral asymmetry index (%BAI) proposed by Impellizzeri and associates [34] and modified in this study based on previous research [9], as shown in the following equation:

$$\%BAI=\left(\frac{Dominant\;side-Non\;dominant\;side}{Dominant\;side}\right)\times 100$$

The Shapiro–Wilk test was applied to assess the normality of these relative differences. Variables whose differences did not follow a normal distribution were analyzed via nonparametric tests. The Wilcoxon test was used to compare the medians of the differences within groups, and the Mann–Whitney *U* test was used to compare the medians between groups, considering a significance level (alpha) of 0.05.

## 3. Results

Table 1 presents the descriptive statistics of the training time and unilateral variables assessed in the canoeist and kayaker groups. Both groups have an average of two years of sports practice and spend 10.7 h per week training.

Table 2 presents the side-by-side analysis of the canoeing and kayaking groups. The hemibodies of the paddlers in both groups showed homogeneity in variables describing their skeletal size in terms of length and breadth (*p* > 0.05). The only significant asymmetries were found in the subscapular skinfold (*p* = 0.010) and the flexed and tensed arm girth in canoeists (*p* = 0.055). For the kayakers, differences between hemibodies were observed only in the mid-thigh girth (*p* = 0.041).

In evaluating the differences between the groups (canoeing versus kayaking) on a single side, it was found that both disciplines were homogeneous in most anthropometric variables when comparing each side (*p* > 0.05). However, the kayakers differed from the canoeists in the subscapular skinfold (*p* = 0.010) and the bicep skinfold (*p* = 0.038) on the right side, whereas on the left side, the differences were limited to the subscapular skinfold (*p* = 0.010).

**Figure 1:**
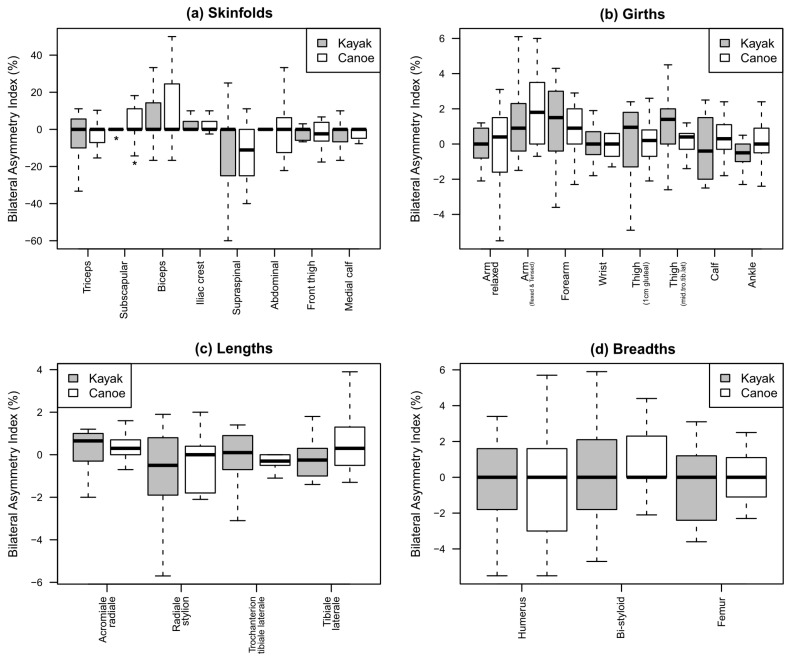
Bilateral asymmetry indices (%BAI) for skinfolds, girths, lengths, and breadths. Box plots marked with an asterisk (*) indicate statistically significant differences, considering a significance level of α = 0.05.

Figure 1 shows the direction of the asymmetries found via the %BAI. The specific observations for each type of measurement are as follows.

Graph (a): Asymmetry trends in skinfolds. Compared with kayakers, canoeists presented marked rightward asymmetry in their subscapular and bicep skinfolds. Kayakers had a higher %BAI in their triceps skinfold. The average values for the rest of the variables were relatively similar. Canoeists had higher extreme values for their subscapular, bicep, and abdominal skinfolds, whereas kayakers had lower extreme values for their tricep and suprailiac skinfolds.

Graph (b): Asymmetry trends in girths. Compared with kayakers, canoe paddlers presented with an average rightward asymmetry in their relaxed arm, flexed and tensed arm, and ankle girths. Kayak paddlers had a higher index for their maximum and middle-thigh girths. The average values for the remaining indices were relatively similar. Canoeists presented higher extreme values for their maximum thigh, whereas kayakers presented higher extreme values for their arm, forearm, and thigh girths.

Graph (c): Asymmetry trends in lengths. Kayak paddlers presented greater heterogeneity in their acromial-radial, radial-stylion, and trochanterion tibial lateral lengths. Canoe paddlers presented higher extreme values for their thigh length (1 cm gluteal), whereas kayakers presented higher extreme values for their acromial-radial, radial-stylion, and trochanterion tibial lateral lengths.

Graph (d): Asymmetry trends in breadths. The average index values are similar for all three breadths in both groups. The asymmetry index for the humerus breadth is more heterogeneous among canoeists, whereas for kayakers, the asymmetry indices of the styloid and femur breadths are more heterogeneous.

Interestingly, the observed asymmetries were not uniform across all participants, by reflected individual variations. These differences suggest that future research could explore how intrinsic factors (e.g., genetics, growth) and extrinsic factors (e.g., training intensity) contribute to these asymmetries.

### Additional Analyses

In addition to the main comparisons, further analyses were conducted to explore possible interactions and differences within demographic subgroups. The role of age in the observed differences was investigated, and no significant interactions were found (*p* = 0.761). The technical error of measurement (TEM) was 5.36% for skinfolds and 2.07% for the remaining variables, indicating a high level of reliability in the anthropometric assessments. A sensitivity analysis was also performed to assess the robustness of the results against variations in measurement methods and participant inclusion criteria. The findings remained consistent (*p* = 0.121), supporting the internal validity of the observed differences between the right and left sides in the anthropometric measurements.

## 4. Discussion

### 4.1. Key Findings

The main objective of this study was to assess the differences in asymmetry between canoeists and kayakers using anthropometric measurements, focusing on the possible differences derived from the asymmetrical nature of canoeing and the more symmetrical demands of kayaking. On the right side of the body, kayakers showed significant differences from canoeists, specifically in the subscapular skinfold and bicep skinfold. In contrast, on the left side, differences were limited to the subscapular skinfold. These asymmetries may reflect sport-specific adaptations. These findings are consistent with previous studies indicating that repetitive movement patterns in asymmetrical sports often lead to localized imbalances in muscle and soft tissue development [35]. Additionally, the absence of significant skeletal asymmetry highlights that these adaptations are more likely to occur in soft tissues than in bone structure during adolescence [36]. Some studies have reported similarities and differences with the present research, although many were conducted with athletes from other sports [24,27,37]. For instance, one study found that football players exhibited significant asymmetries in their knee extensors and flexors, especially in the U13 and U15 categories, but these asymmetries diminished in older categories (U17). These findings suggest that long-term adaptations and balanced training can mitigate initial asymmetries [37]. In our research, although the motor gestures in canoeing are asymmetrical, young paddlers did not show significant differences in their anthropometric profiles, which may be due to early adaptations and balanced training programs. This finding supports the idea that asymmetries in young athletes are often transient and respond to well-structured training interventions [38].

Our initial hypothesis predicted a greater number of asymmetries due to the biomechanical nature of each discipline. Existing studies suggest that canoeing is an asymmetrical sport due to the unilateral movements performed during paddling, while kayaking is considered more symmetrical, as it uses both sides of the body more evenly [13,29]. However, contrary to this expectation, no other significant differences were observed between canoeists and kayakers beyond those that have already been mentioned.

Another key finding was that canoeists had less experience practicing the sport (1.4 years) and fewer weekly training hours (9 h) compared to kayakers (2.6 years and 12.2 h). However, despite this significant difference in experience, no characteristic pattern emerged when comparing the right and left sides of the body between the two modalities. These results align with a study conducted by Saal and associates, which suggests that athletes may exhibit limited asymmetries during their developmental phase, highlighting the importance of balanced training programs to address emerging imbalances [38].

Overall, no significant differences were found between canoeists and kayakers in terms of symmetry; this study highlights the potential of anthropometry, as a highly reproducible tool, for monitoring possible asymmetries over time. The findings suggest that regular anthropometric evaluations could be invaluable for assessing the effectiveness of training programs and preventing the development of asymmetries that could increase the risk of long-term injuries. Additionally, these results support the idea that asymmetries in young athletes may not be significant in the early stages of training but could develop over time as they continue their practice and specialize in their sport.

### 4.2. Limitations

The main limitation of this study is the sample size, as no prior calculation was made, which may have affected its statistical power in detecting significant differences. Future studies with larger samples could provide more robust and generalizable results.

Another relevant limitation is the significant difference in sports experience between the groups analyzed. On average, the kayaking group had 1.2 more years of experience than the canoeing group, which may have influenced the results and prevented an equitable comparison. Evaluating adolescents with a broader spectrum of experience in both sports could help us understand how muscular and structural adaptations affect asymmetry and to what extent balanced training programs and technical experience can reduce these differences [13,16,29]. A recent study revealed that young athletes practicing asymmetrical sports, such as tennis and volleyball, showed greater asymmetries compared to those participating in symmetrical sports like triathlon and gymnastics. However, these differences were more pronounced in athletes with more years of experience [24]. Although it may seem contradictory, the lack of significant asymmetries in athletes with few years of experience suggests that asymmetries develop over time and with sport specialization. Therefore, early monitoring and corrective measures play a key role in minimizing excessive asymmetries [25].

Additionally, another important factor is the possible influence of growth and puberty on anthropometric characteristics, as adolescents undergo significant physical changes during their maturation. Factors such as the onset of puberty and growth spurts could have affected the observed asymmetries. Since this study did not assess these developmental factors, it is not possible to fully determine their influence on the results. Ramos-García and colleagues [39] highlight the importance of considering growth in anthropometric studies of adolescents, suggesting that future research that includes growth assessments could provide a more comprehensive understanding of how maturation affects the development of asymmetry in young athletes.

It is important to note that biological maturation does not always follow a linear pattern [22,39], so its impact on the development of asymmetries may vary among individuals. Future studies could benefit from the inclusion of biological age assessments or maturation analyses to better understand these effects and distinguish between changes attributable to natural development and those derived from specific training.

In relation to the points previously discussed, Krzykała and colleagues [13] noted that young canoe athletes, due to specialized training, may develop sport-specific asymmetries during their biological development. However, in our study population, this asymmetry did not manifest, possibly due to the reduced sample size (previously mentioned as the main limitation of this study) and the relatively shorter sports experience of the canoeing group compared to the kayaking group.

An important limitation of this study is the lack of a longitudinal analysis, which would have allowed for observing the evolution of asymmetries over time, and another is the absence of asymmetry assessments using advanced complementary tools, such as three-dimensional imaging and biomechanical analyses. These tools could have provided a more detailed view of how technique and movement mechanics influence the development of asymmetries. A longitudinal approach would allow for evaluating how asymmetries evolve across different stages of sports development and establish whether there are critical moments at which an intervention could be more effective. Additionally, future studies could focus on key variables such as training load progression and sports specialization to determine their impact on the emergence and magnitude of asymmetries.

In line with this perspective, a longitudinal study has suggested that asymmetries may evolve depending on the training load and competitive level, with more experienced athletes typically exhibiting more refined biomechanical adaptations [21]. This highlights the importance of structured intervention programs, which could not only correct imbalances but also improve performance by leveraging controlled asymmetries. Specifically, in water sports, integrating biomechanical analyses into training protocols can provide valuable information to optimize stroke mechanics and minimize the risk of injury [27], which is essential and allows coaches to design more specific and effective interventions [29]. In this context, the use of advanced tools (such as those mentioned above) would have increased our accuracy in detecting asymmetries in our study population.

### 4.3. Interpretation

Several studies suggest that asymmetrical differences between young and adult athletes can be attributed to factors such as muscle development, technique, long-term adaptations, and injury history, reflecting how the body adapts to the specific demands of a sport over time [13,16,25,26]. The absence of significant asymmetries in this study may be due to the athletes still being in an early stage of their development, where training adaptations have not yet fully consolidated. At this phase, any imbalances may be transient, as the body is still in the process of adjusting and maturing.

Longitudinal studies have suggested that asymmetries may evolve based on the training load and competitive level of the athlete, with more experienced athletes often exhibiting more refined biomechanical adaptations [21]. This finding emphasizes the potential of structured intervention programs, not only to address imbalances but also to enhance performance by leveraging controlled asymmetries. Specifically, in water sports, integrating biomechanical analyses into training protocols could provide useful insights to optimize stroke mechanics and minimize the risk of injury [27].

Although both groups engage in intense physical activity, the specific demands of each discipline may induce slightly different adaptations in certain body regions. The few significant differences observed in the subscapular and bicep skinfolds on the right side, as well as in the subscapular skinfold on the left side, suggest that although no significant asymmetries were found overall, each sport may generate subtle morphological variations (as previously mentioned). These differences could be related to rowing technique, muscle activation patterns, and the load distribution in each discipline.

Recent studies highlight the importance of considering the specific mechanical demands of each sport when interpreting asymmetry data. It has been proposed that sports with unilateral gestures, such as canoeing, may induce compensatory adaptations that do not necessarily negatively affect performance [23]. In fact, these adaptations could optimize athlete efficiency, even in the presence of asymmetries, especially during the early stages of sports development. However, further research is still needed to determine whether these adaptations offer performance advantages or whether they pose long-term risks [24].

### 4.4. Generalization

The generalization of these results should be made with caution. Although our findings align with previous studies in suggesting that asymmetries may not be significant in the early stages of training [24,27], other works have reported marked asymmetries in young athletes [13,23,37]. This indicates that the emergence of asymmetries could depend on various factors, such as the specific demands of the sport, the duration and intensity of training, and the individual characteristics of the athletes. These factors could significantly influence the manifestation of asymmetries. To better understand these aspects, it would be helpful to conduct studies with larger and more diverse samples. Furthermore, additional variables such as training techniques, genetic predisposition, and injury history should be considered when evaluating the generalization of the results, as they may play an important role in the observed differences.

### 4.5. Relevance and Practical Applications

The continuous assessment of asymmetries using more reproducible tools, such as anthropometry, could directly impact the development of balanced training programs aimed at mitigating potential asymmetries, thereby improving the performance and health of athletes.

In summary, these findings provide a valuable foundation for understanding the asymmetries in young canoeists and kayakers. However, further studies are needed to generalize these results to other populations and sporting contexts. Understanding at what point asymmetries cease to be functional and become risk factors could be key to designing specific interventions [14]. As mentioned in the Introduction, most studies in this field have used a limited number of anthropometric indicators to assess asymmetry differences [1,5,19,20,28]. In this regard, the present study is one of the first to address these differences using a broad and detailed anthropometric profile, making it an important reference for future research in this field.

## 5. Conclusions

This study identified significant differences in the anthropometric asymmetry profiles of adolescent canoeists and kayakers, but these differences were observed in only 3 of the 58 variables analyzed. These findings suggest that the differences were more limited and less pronounced than initially expected. These results highlight the need for further research to examine the relationship between sports experience and asymmetries, with the aim of identifying adaptive patterns in aquatic sport disciplines. A better understanding of these patterns could help optimize the design of balanced training plans aimed at supporting performance and reducing injury risk, with an emphasis on the importance of monitoring asymmetries from an early stage.

Furthermore, this study reinforces the value of anthropometry as a highly reproducible tool for monitoring asymmetries in young athletes. Future research integrating this with long-term monitoring and/or biomechanical analyses could provide deeper insights into these adaptations, offering practical applications for coaches and sports professionals.

## Figures and Tables

**Table 1:** General characteristics of the study sample and their unilateral anthropometric variables, including the differences between canoeists and kayakers.

	Variable	Canoe (*n* = 13)	Kayak (*n* = 14)	p *
		Median (IQR)	Median (IQR)	
General information	Age (years)	13.0 (2.0)	13.0 (2.0)	1.000
	Length of practice (years)	1.0 (5.0)	1.75 (6.5)	0.023 *
	Weekly training (h)	10.0 (9.5)	12.0 (21.5)	0.058
Basics	Body mass (kg)	56.2 (32.2)	50.8 (46.8)	0.452
	Stature (cm)	160.4 (25.1)	165.2 (18.8)	0.182
	Sitting height (cm)	79.6 (16.5)	81.3 (15.5)	0.234
	Arm span (cm)	163.6 (28.8)	166.4 (20.7)	0.512
Girths and Breadths	Chest girth (cm)	83.0 (30.5)	79.8 (32.9)	0.716
	Waist girth (cm)	70.2 (26.0)	68.5 (26.9)	0.846
	Hip girth (cm)	85.1 (17.5)	84.0 (31.1)	0.482
	Iliospinale length (cm)	101.5 (16.8)	101.3 (76.8)	0.680
	Biacromial breadth (cm)	29.0 (9.2)	29.5 (15.4)	0.450
	Biiliocristal breadth (cm)	19.8 (11.6)	21.0 (12.6)	0.295
	Transverse chest breadth (cm)	23.2 (7.9)	22.4 (12.5)	1.000
	Antero-posterior chest breadth (cm)	17.0 (8.0)	16.3 (13.0)	0.367

Values are presented as medians and interquartile ranges; * statistical significance according to Mann–Whitney U test (*p* < 0.05).

**Table 2:** Comparison of anthropometric asymmetries between canoe and kayak athletes: intra- and intergroup analysis.

Variable		Canoe (*n* = 13)			Kayak (*n* = 14)			All (*n* = 27)	
		Right Side	Left Side	*p*	Right Side	Left Side	*p*	*p*	*p*
								(Right Side)	(Left Side)
Skinfolds (mm)	Triceps	13.0 (7.6)	13.0 (7.0)	0.784	9.2 (4.2)	9.0 (4.2)	0.951	0.113	0.084
	Subscapular	9.0 (6.0)	9.0 (6.0)	0.275	6.5 (2.0)	7.0 (2.0)	1.000	0.010 **	0.010 **
	Biceps	7.0 (6.0)	5.0 (6.0)	0.143	4.0 (3.0)	4.0 (2.0)	0.203	0.038 *	0.117
	Iliac crest	12.0 (11.0)	12.0 (8.0)	0.240	10.0 (5.8)	9.5 (6.0)	0.203	0.188	0.158
	Supraspinal	9.0 (9.0)	10.0 (8.0)	0.017 *	6.5 (7.5)	7.8 (6.2)	0.256	0.224	0.144
	Abdominal	11.0 (8.0)	10.0 (6.0)	0.916	8.5 (4.1)	8.5 (4.9)	0.766	0.273	0.263
	Front thigh	16.0 (6.0)	17.0 (7.0)	0.120	14.5 (4.2)	15.0 (4.8)	0.457	0.067	0.061
	Medial calf	14.0 (8.0)	14.0 (8.0)	0.386	12.5 (4.8)	12.5 (5.8)	0.390	0.196	0.188
Girths (cm)	Arm (relaxed)	26.0 (4.1)	25.9 (4.7)	0.674	24.0 (1.5)	24.4 (0.9)	1.000	0.382	0.356
	Arm (flexed and tensed)	27.9 (4.6)	27.9 (5.9)	0.055 *	26.8 (2.0)	26.8 (1.7)	0.131	0.716	0.716
	Forearm	23.5 (2.5)	23.0 (1.8)	0.655	23.4 (1.4)	23.6 (1.1)	0.123	0.734	0.644
	Wrist	15.6 (1.0)	15.5 (0.9)	0.412	15.6 (0.8)	15.4 (0.9)	0.535	0.752	0.808
	Thigh (1 cm gluteal)	52.2 (9.0)	52.1 (7.8)	0.834	50.0 (2.9)	49.2 (1.2)	0.379	0.275	0.344
	Mid-thigh	48.0 (7.7)	47.4 (8.3)	0.272	44.2 (2.6)	44.1 (2.8)	0.041 *	0.452	0.409
	Calf	34.0 (5.9)	34.6 (4.9)	0.125	33.0 (2.3)	33.3 (3.0)	0.777	0.734	0.846
	Ankle	21.0 (3.0)	22.3 (3.0)	1.000	21.8 (0.9)	22.0 (1.8)	0.148	0.808	0.884
Lengths (cm)	Acromiale-radiale	30.3 (3.4)	30.5 (3.1)	0.326	30.4 (1.6)	30.4 (1.8)	0.344	0.903	1.000
	Radiale-stylion	24.5 (3.1)	24.0 (2.6)	0.258	25.4 (3.8)	25.3 (4.3)	0.419	0.308	0.331
	Trochanterion tib.lat	37.4 (7.6)	38.0 (6.4)	0.448	44.1 (5.2)	43.8 (5.1)	0.889	0.094	0.120
	Tibiale laterale	37.3 (2.5)	37.5 (2.2)	0.600	37.2 (2.9)	37.3 (2.4)	0.220	1.000	1.000
Breadths (cm)	Humerus	5.9 (0.7)	5.9 (0.7)	0.124	5.8 (0.6)	5.8 (0.6)	0.811	0.769	1.000
	Bistyloid	4.6 (0.6)	4.6 (0.6)	0.573	4.8 (0.4)	4.8 (0.4)	0.719	0.306	0.329
	Femur	8.7 (0.6)	8.7 (0.6)	0.723	8.5 (0.6)	8.6 (0.3)	0.608	0.465	0.480

Values are presented as medians and interquartile ranges; * statistical significance according to Wilcoxon signed-rank test or Mann–Whitney U test (*p* < 0.05); ** statistical significance according to Mann–Whitney U test (*p* < 0.01).

## Data Availability

The original contributions presented in this study are included in the article. Further inquiries can be directed to the corresponding author(s).

## Abbreviations

The following abbreviations are used in this manuscript:

DXA	Dual-energy X-ray absorptiometry
BIA	Body Electrical Impedance Analysis
STROBE	Strengthening the Reporting of Observational Studies in Epidemiology
TEM	Technical error of measurement
%BAI	Bilateral asymmetry index
